# Exploration of the Therapeutic Time Window for Thrombectomy in Rat Models of Middle Cerebral Artery Ischemia‐Reperfusion

**DOI:** 10.1002/brb3.70713

**Published:** 2025-08-04

**Authors:** Yuting He, Pengcheng Huang, Shumeng Li, Jing Lin, Xiao Ren, Ying Xiong, Yuanjian Yang, Daojun Hong

**Affiliations:** ^1^ Department of Neurology, The First Affiliated Hospital, Jiangxi Medical College Nanchang University Nanchang China; ^2^ Institute of Neurology, Jiangxi Academy of Clinical Medical Science, The First Affiliated Hospital, Jiangxi Medical College Nanchang University Nanchang China; ^3^ Rare Disease Center the First Affiliated Hospital of Nanchang University Nanchang China; ^4^ Medical Experimental Center Jiangxi Mental Hospital/Affiliated Mental Hospital of Nanchang University Nanchang China; ^5^ Department of Psychiatry Jiangxi Mental Hospital/Affiliated Mental Hospital of Nanchang University Nanchang China; ^6^ Jangxi Provincial Clinical Research Center on Mental Disorders Nanchang China; ^7^ Key Laboratory of Rare Neurological Diseases of Jiangxi Provincial Health Commission the First Affiliated Hospital of Nanchang University Nanchang China

**Keywords:** blood–brain barrier, cerebral ischemia, reperfusion injury, thrombolysis

## Abstract

**Objective:**

To investigate the therapeutic time window of middle cerebral artery occlusion (MCAO) reperfusion in rats and the potential mechanism of injury beyond the time window.

**Methods:**

Male Sprague Dawley (SD) rats were randomly divided into eight groups: a sham operation group, six ischemia‐reperfusion groups (reperfusion initiated 1, 2, 3, 4, 5, and 6 h after infarction, respectively), and a 24‐h infarction group without reperfusion therapy. Neurological function scores were assessed 24 h after reperfusion, and then brain samples were collected to explore the mechanisms.

**Results:**

After 4 h of MCAO reperfusion for 24 h, the infarct area reached its peak, while the neurological function score reached its bottom, and both indicators exhibited a trend toward stabilization. Additionally, a significant increase in the mortality rate was observed in the 5‐h group. Compared to the MCAO 2‐h group, the 5‐h group exhibited a significant increase in the number of dead neurons, more serious disruption of the blood–brain barrier (BBB), and elevated expression levels of IL‐6, IL1‐β, and TNF‐α.

**Conclusion:**

Taken together, our study indicates that MCAO 4 h is likely to be the therapeutic time window for thrombectomy in rat models of middle cerebral artery ischemia‐reperfusion. Reperfusion injury beyond the time window leads to more severe disruption of the BBB, a significant increase in inflammatory products, and may ultimately result in a significant decline in neural function scores.

## Graphical abstract

MCAO 4 hours is likely to be the therapeutic time window for thrombectomy in rat models of MCA ischemia‐reperfusion. Reperfusion injury beyond the time window leads to more severe disruption of BBB, an significant increase in inflammatory products, and may ultimately result to a significant decline in neurological function.

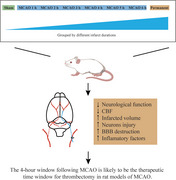

## 1 Introduction

Acute ischemic stroke (AIS) is a severe condition resulting from impaired blood circulation, leading to severe neurological impairment and potential mortality. During the acute period of AIS, recanalization therapy has emerged as the primary treatment modality, with the time window for mechanical thrombectomy expanding from 6 to 16 or even 24 h according to guidelines (Albers et al. 2018; Campbell and Khatri 2020; Nogueira et al. 2018). Mechanical thrombectomy achieves vascular recanalization in over 90% of AIS patients; however, approximately half of these patients encounter unfavorable clinical outcomes (Heit et al. 2021). Research has demonstrated that early thrombectomy therapy results in a significantly higher rate of favorable outcomes compared to those who receive thrombectomy therapy at a later stage (Mundiyanapurath et al. 2017). Therefore, the significance of thrombectomy beyond a certain time limit is worthy of further exploration. However, due to ethical and practical limitations, this part of the experiment is difficult to conduct with human subjects, so we opted for fundamental experiments to explore this issue. However, we are confronted with the challenge of the absence of thrombectomy‐reperfusion injury models or paradigms that account for varying infarction durations.

Previous studies have demonstrated that cerebral ischemia‐reperfusion injury (CIRI) is involved in a diverse array of intricate physiological and pathological mechanisms, comprising the disruption of the blood–brain barrier (BBB), inflammatory response, oxidative stress, and mitochondrial dysfunction (Ajoolabady et al. 2021). However, there has been no comprehensive study on animal models of ischemia‐reperfusion to determine the therapeutic time window for thrombus extraction, and the underlying pathophysiological mechanisms beyond the time window remain unclear. Therefore, exploring the therapeutic time window for thrombectomy and its underlying pathological mechanisms in ischemia‐reperfusion rats holds practical significance.

This study established the middle cerebral artery occlusion (MCAO) model in Sprague Dawley (SD) rats, followed by reperfusion of blood flow at various time points post‐embolization. The alterations in neurological function, infarct volume, BBB structure and function, as well as inflammatory factors, were investigated at different time points following ischemia‐reperfusion. The object of this study is to establish an experimental foundation for identifying the therapeutic time window in rat models of ischemia‐reperfusion and to evaluate potential mechanisms underlying reperfusion injury beyond the time window. This provides a theoretical foundation for further research into the mechanisms of ischemia‐reperfusion injury and the development of related neuroprotective drugs.

## 2 Materials and Methods

### 2.1 Animals

A total of 150 male SD rats (weight, 250–280 g) were used for the experiments. These rats were accommodated in a well‐ventilated environment with access to sufficient water and feed, following a 12‐h light–dark cycle. All experimental procedures were conducted in strict adherence to the relevant ethical regulations and were approved by the Experimental Animal Welfare Ethics Committee at the First Affiliated Hospital of Nanchang University (Permission CDYFY‐IACUC‐202312QR004).

### 2.2 Establishment of Rat MCAO Model

The animals were divided into eight groups at random: a sham operation group (sham, *n* = 24), a permanent cerebral ischemia group (permanent, *n* = 24), and six ischemia‐reperfusion groups with threads removed at six different time points (1 h, *n* = 24; 2 h, n = 32; 3 h, *n* = 17; 4 h, *n* = 17; 5 h, *n* = 32; and 6 h, *n* = 17) after thread insertion. The neurological function was evaluated after a reperfusion treatment period of 24 h, and then the rats were sacrificed. The animals underwent an 8‐h dietary restriction and were then anesthetized with isoflurane (3%–4% induction, 2% maintenance). The surgical site was sterilized with 75% alcohol. A midline incision was made in the neck to expose the right common carotid artery (RCCA), internal carotid artery (ICA), and external carotid artery (ECA), while carefully separating them from adjacent nerves and tissues. The ECA was ligated, and a thread with a silicon tip (Rayward, A5‐243650) was inserted into the ECA stump approximately 18–22 mm to occlude the RMCA. The sham group and permanent group underwent similar procedures, except for the absence of arterial embolization in the sham group and thread removal in the permanent group.

### 2.3 Neurological Function Assessment

The improved Garcia JH scoring system (Garcia et al. 1995) was utilized to evaluate the neurological function of all rats at 24 h post‐surgery, and the scoring contents lay in the focus on six aspects: autonomic movement, body symmetry, forelimb extension function, climbing movement, bilateral body touch, and bilateral beard touch (Table S1). Scores ranged from 3 to 18 points, with higher scores indicating less neurological impairment.

### 2.4 Cerebral Blood Flow Measurement by Laser Speckle Contrast Imaging

The rats were anesthetized with isoflurane, and their scalps were sterilized with 75% alcohol. A longitudinal incision was then made to expose the skull. Next, the skull was carefully polished using a rasp. Finally, it was gently wiped with a cotton ball soaked in normal saline solution to maintain moisture. The rats were then placed under the laser speckle contrast imaging (LSCI) system (SIM BFI ZOOM Pro, SIM Opto‐Technology Co. Ltd, Wuhan, China) to monitor cerebral blood flow (CBF) images. A computer algorithm was used to convert the velocity information of blood flow into a two‐dimensional flow map. The images were performed reverse color processing using the software provided by the system, and the data was analyzed on these images. CBF images were obtained at baseline, immediately after embolization, prior to reperfusion (random time points for the sham group and the permanent group, and before thread removal for the reperfusion group), immediately after thread removal, and 24 h after reperfusion. Some regions of interest (ROIs) were selected to measure CBF. The ROIs in the ischemic hemisphere were labeled from top to bottom as ROI 1, ROI 3, and ROI 5, while ROIs in the contralateral hemisphere were labeled as ROI 2, ROI 4, and ROI 6. The rCBF ratio was calculated as the percentage of ischemic CBF relative to contralateral CBF (ROI 1/ROI 2, ROI 3/ROI 4, and ROI 5/ROI 6).

### 2.5 TTC Staining

The rats were deeply anesthetized with pentobarbital sodium (50 mg/kg) and subsequently infused with 0.01 M phosphate‐buffered saline (PBS). Immediately after perfusion, brains were carefully removed and then frozen at −20°C for 20 min and cut into coronal slices at a thickness of approximately 2 mm. Then, brain slices were incubated with 2% TTC (w/v) for 20 min in a 37°C water bath, avoiding light. After being fixed in 4% paraformaldehyde for 12 h, the slices were scanned. The infarct regions appear as white, whereas the non‐infarct areas exhibit a red coloration. Image‐Pro Plus 7.0 software was used to analyze the infarct area; infarct ratio (%) = right infarct area/right total area × 100%.

### 2.6 Hematoxylin and Eosin Staining

The brain tissues were prepared, fixed, sliced and then preserved at room temperature. Hematoxylin and Eosin (HE) staining was performed according to the following procedure. The slices were sequentially immersed in xylene for 15 min, followed by ethanol of gradient concentrations (100%, 95%, 90%, 80%, and 70%) for 10 min for each process. The slices were then stained with hematoxylin for 8 min, followed by differentiation in a solution of 1% hydrochloric acid alcohol for a few seconds, and then washed after the color of the slices changed. Eosin staining was performed for an additional duration of 3 min, followed by dehydration and transparency using anhydrous ethanol and xylene, respectively. Subsequently, the dried slices were sealed with neutral resin before observing brain histopathological changes under a light microscope and collecting images.

### 2.7 Nissl Staining

Nissl staining was utilized to observe neuronal morphological alterations 24 h after reperfusion (*n* = 5). The procedures adhered to were performed according to the specifications of the Nissl staining kit (C0117, Beyotime, China). The Nissl‐positive neurons in the penumbra were observed blindly via a light microscope (HS6; Ningbo Sunny Instruments Co. Ltd, China).

### 2.8 Immunohistochemical Staining

The brains were fixed in 4% paraformaldehyde for a night at 4°C and sectioned with a vibratome (Leica, VT1000s) at a thickness of 35 µm. Then, the sections were incubated with an endogenous peroxidase blocker (SPN‐9001, Beijing Zsbio) for 30 min. Primary antibodies used included IgG (A7031, Shanghai Beyotime), IL‐1β (66737‐1‐Ig, Wuhan Proteintech), TNF‐α (60291‐1‐Ig, Wuhan Proteintech), and IL‐6 (21865‐1‐AP, America Thermo Fisher). The sections were incubated overnight with the primary antibody at 4°C. Enzyme‐labeled goat anti‐mouse/rabbit IgG polymer (SPN‐9001, Beijing Zsbio) was then incubated at 37°C for 30 min. Finally, diaminobenzidine (DAB, ZLI‐9017, Beijing Zsbio) was employed for chromogenic development. Image‐Pro Plus 7.0 software was utilized to measure the exosmotic area of IgG and the total positive area of inflammatory cells on the infarction side.

### 2.9 Western Blot Analysis

The proteins were extracted from the infarcted hemisphere of rats using Radio Immunoprecipitation Assay lysis buffer (Shanghai Beyotime) with Halt Protease and transferred to a PVDF membrane through the wet transfer method employing SDS‐PAGE electrophoresis. The membrane was then incubated with primary antibodies against ZO‐1 (61‐7300, America Thermo Fisher), occludin (27260‐1‐AP, Wuhan Proteintech), CD31 (553369, America BD Biosciences), and GAPDH (30202ES60, Shanghai Yeasen Biotechnology) at 4°C overnight. Subsequently, the membrane was incubated with secondary antibodies, including goat anti‐mouse IgG (511103, Chengdu Zen Bioscience) and goat anti‐rabbit IgG (511203, Chengdu Zen Bioscience), for 2 h at room temperature, followed by ECL development. Finally, the grayscale intensity of each band was analyzed by Image‐Pro Plus 7.0 software.

### 2.10 Statistical Analysis

The data were analyzed using GraphPad Prism 9 software, and those conforming to the normal distribution were expressed as mean ± standard error of the mean (SEM). The measurement data was analyzed by using the chi‐square test, one‐way, and two‐way analysis of variance. A significance level of *p* < 0.05 was considered statistically significant.

## 3 Result

### 3.1 The Therapeutic Time Window of Thrombectomy in Rats Was Within 4 h After MCAO

**FIGURE 1:**
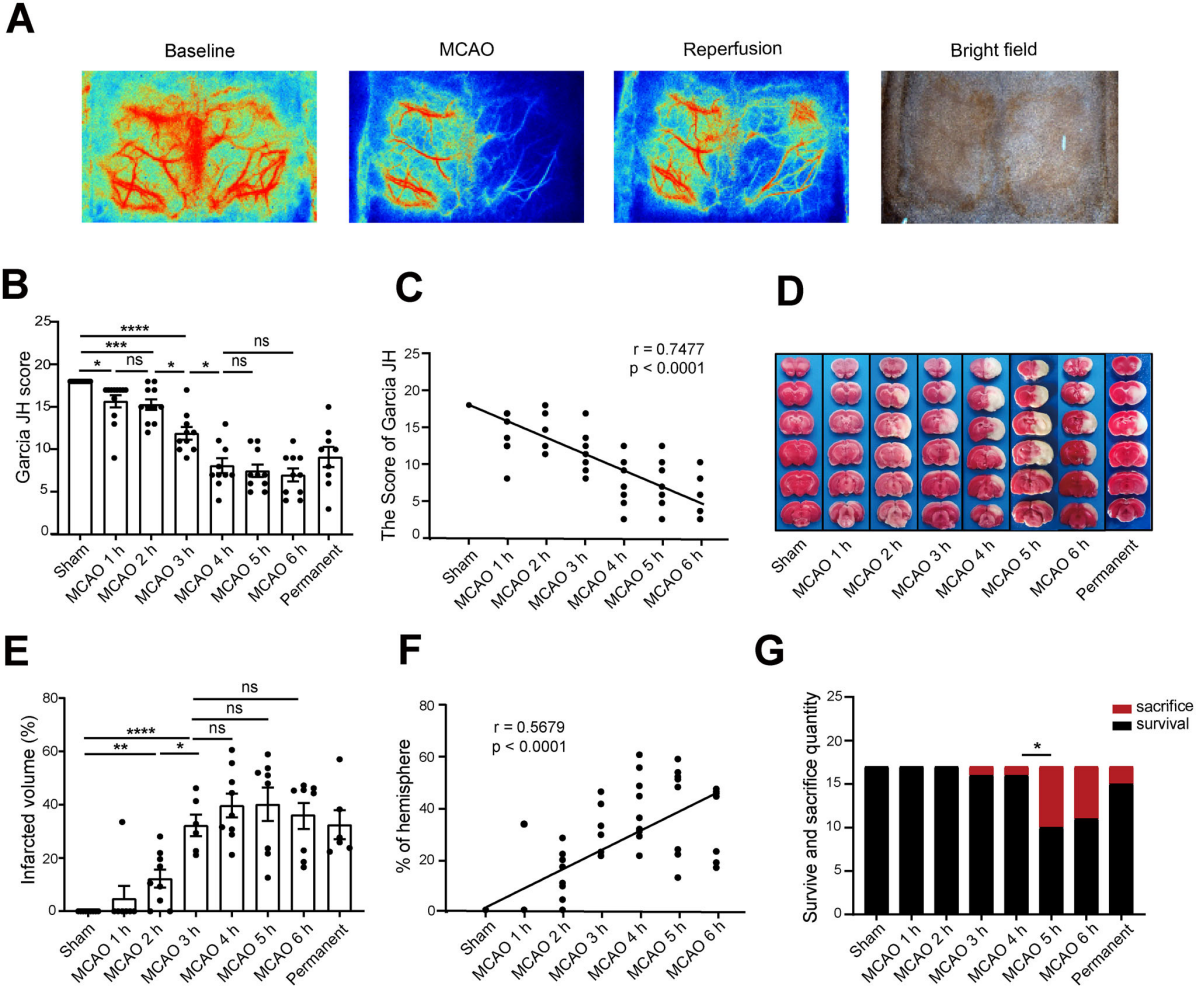
The time window for thrombectomy after MCAO is 4 h in rats, and neurological deficit in this timeframe correlated with infarct volume. (A) Representative laser speckle images showing CBF at pre‐operation (baseline), MCAO, and reperfusion. (B) Neurological deficit score. *n* = 9–12, data are presented as the mean ± S.E.M. *p < 0.05, one‐way ANOVA followed by Bonferroni post hoc test. (C) The scatter plot shows a positive correlation between infarct duration and Garcia JH score. *n* = 9–12, *p < 0.0001, linear regression correction approach. (D) TTC staining of brain slices from each group of rats at 24 h after MCAO. The white area was defined as the infarct area. (E) Brain infarct volumes were quantified. *n* = 6–8, data are presented as the mean ± S.E.M. *p < 0.05, one‐way ANOVA followed by Bonferroni post hoc test. (F) The scatter plot shows a negative correlation between infarct duration and infarct volumes. *n* = 6–8, *p < 0.0001, linear regression correction approach. (G) Survival and sacrifice quantity within 24 h after the operation. *n* = 17, chi‐square test was used for the data, *p < 0.05.

We used LSCI to monitor the effects of MCAO and reperfusion during the operation. Once MCAO was started, CBF in the right cerebrum markedly decreased and recovered slightly after removal of the thread (Figure 1A, Figure S1), which verified the success of the MCAO modality. To investigate the therapeutic time window for thrombectomy in rat models, the Garcia JH score was used to assess neurological function. The results showed that the Garcia JH score was negatively correlated with the duration of infarction; that is, the Garcia JH score decreased gradually with the increase of infarction duration (Figure 1B,C) and a significant decrease in the neurological function in the MCAO 3‐h group, reaching its lowest point in the MCAO 4‐h group, and exhibited a trend toward stabilization (Figure 1B). The infarct volume was evaluated through TTC staining. We observed a positive correlation between the duration of MCAO and the volume of infarct, as depicted in Figure 1D–F. Statistical analysis revealed that the infarct volume significantly increased in the MCAO 3‐h group and reached its peak in the MCAO 4‐h group (Figure 1E). However, further analysis of mortality revealed that the MCAO 4‐h group did not result in a significant increase in mortality compared with the MCAO 3‐h group. However, a significant increase in mortality was observed in the MCAO 5‐h group (Figure 1G). Therefore, compared to the MCAO 5‐h group, thrombectomy reperfusion in the MCAO 4‐h group did not significantly slow the deterioration of neurological function; however, it substantially reduced the mortality rate. Collectively, neurological impairment in rats is exacerbated with prolonged ischemia time, while the therapeutic time window for effective thrombectomy in rats appears to be within 4 h after MCAO.

### 3.2 The Prognosis of Thrombectomy Beyond the Time Window Was Inferior Compared With That Within the Timeframe

**FIGURE 2:**
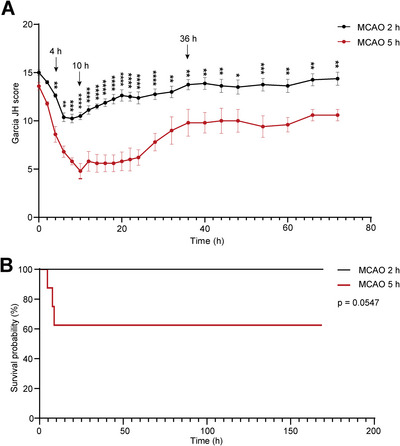
The prognosis of thrombectomy beyond the time window was inferior compared to that within the timeframe. (A) Neurological function scores of rats in the MCAO 2‐h group and 5‐h group were assessed within 72 h after thrombectomy. *n* = 8, data are presented as the mean ± S.E.M. *p < 0.05, **p < 0.01, ***p < 0.001, ****p < 0.0001, two‐way ANOVA followed by Sidak post hoc test. (B) The survivorship curve of rats in the MCAO 2‐h group and 5‐h group within 1 week after thrombectomy, *n* = 8.

In previous studies, thrombectomy in MCAO SD rats has usually been performed at 2 h after infarction, which has been identified as the therapeutic duration of ischemia for replicating the ischemia reperfusion model in SD rats (Fluri et al. 2015; Q. Y. Yang et al. 2021). Our studies suggested that the therapeutic time window for thrombectomy in MCAO SD rats was limited to within 4 h postinfarction. Therefore, we selected thrombectomy at the MCAO 2‐h group as the “within‐time‐window” group and thrombectomy at the MCAO 5‐h group as the “out‐of‐time‐window” group for further study, aiming to explore the potential factors that contribute to increased mortality beyond the therapeutic window for thrombectomy. Dynamic evaluations of neurological function were conducted within 72 h after thrombectomy. The neurological score of the 5‐h group demonstrated a significant decline at 4 h after thrombectomy, reaching its lowest point at 10 h. Subsequently, the score gradually improved, plateauing at 36 h and continuing to 72 h, albeit with significant differences persisting between the 5‐h group and the 2‐h group (Figure 2A). The survival rates of both groups were assessed within a week, revealing that 3 out of 8 rats in the 5‐h group died at 4, 7, and 8 h after reperfusion therapy. All the remaining rats (*n* = 5) in the 5‐h group and those (*n* = 8) in the 2‐h group survived for 1 week (Figure 2B). Collectively, compared with thrombectomy within the time window, the prognosis for MCAO rats undergoing thrombectomy beyond the time window was notably worse.

### 3.3 The Recovery of CBF on the Cortex Surface After MCAO Is Not Significantly Affected by Different Ischemia‐Reperfusion Times

**FIGURE 3:**
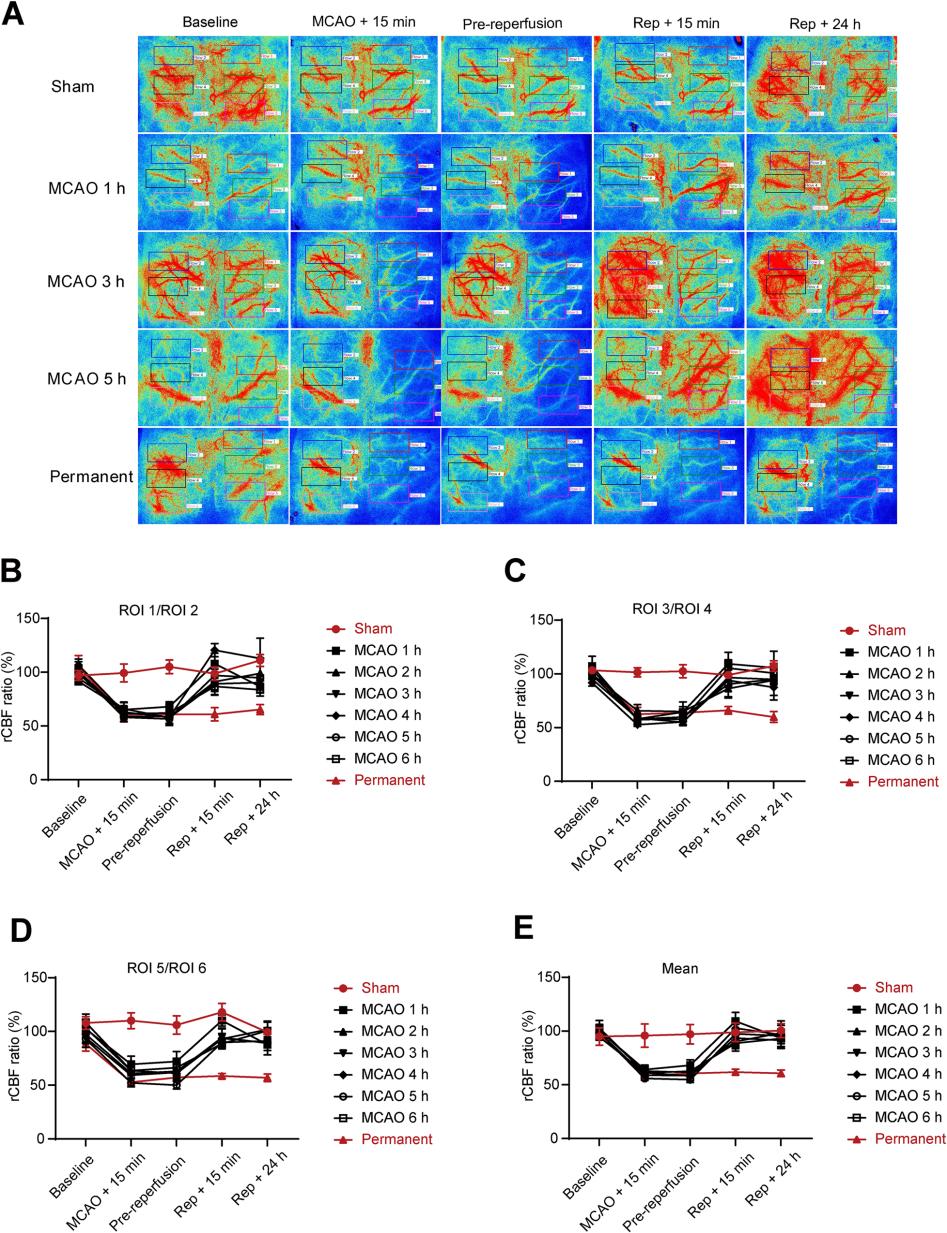
The recovery of cerebral blood flow on the cortex surface after MCAO is not significantly affected by different ischemia‐reperfusion times. (A) Representative laser speckle images showing CBF at pre‐operation (baseline), 15 min after MCAO (MCAO + 15 min), MCAO *n* hours (1, 2, 3, 4, 5, and 6 h, respectively; pre‐reperfusion), 15 min after reperfusion (Rep + 15 min), and 24 h after reperfusion (Rep + 24 h). (B, C, D) Quantification of rCBF in the ischemic hemisphere at each time point. *n* = 5. The ROIs in the ischemic hemisphere were labeled from top to bottom as ROI 1, ROI 3, and ROI 5, while ROIs in the contralateral hemisphere were labeled as ROI 2, ROI 4, and ROI 6. The rCBF ratio (%) was calculated as the percentage of ischemic cerebral blood flow relative to contralateral cerebral blood flow (ROI 1/ROI 2, ROI 3/ROI 4, and ROI 5/ROI 6). (E) Quantified mean values of rCBF in ischemic hemispheres at each time point, that is, (ROI 1/ROI 2 + ROI 3/ROI 4 + ROI 5/ROI 6)/3.

To further evaluate the changes in CBF at different durations of ischemia after MCAO, we used LSCI to record the dynamic changes of CBF in the infarct region before, during, and after surgery. The CBF was measured in eight groups at five time points, including baseline, 15 min after MCAO, before reperfusion, 15 min after reperfusion, and 24 h after reperfusion. Following MCAO, CBF values in all three focal regions we selected of the ischemic hemisphere significantly decreased. However, they gradually recovered after the embolus was removed (Figure 3A). At the same monitoring time point (MCAO + 15 min, pre‐reperfusion, Rep + 15 min, or Rep + 24 h), there was no significant difference observed in the ischemic hemispheric CBF values among the reperfusion groups (Figure 3B–E). The results not only demonstrated the reliability of the MCAO models but also indicated that different ischemia‐reperfusion times would not significantly impact the recovery of CBF on the cortex surface after MCAO.

### 3.4 Reperfusion Injury From Thrombectomy Beyond the Therapeutic Time Window May Aggravate Neuronal Cell Damage

**FIGURE 4:**
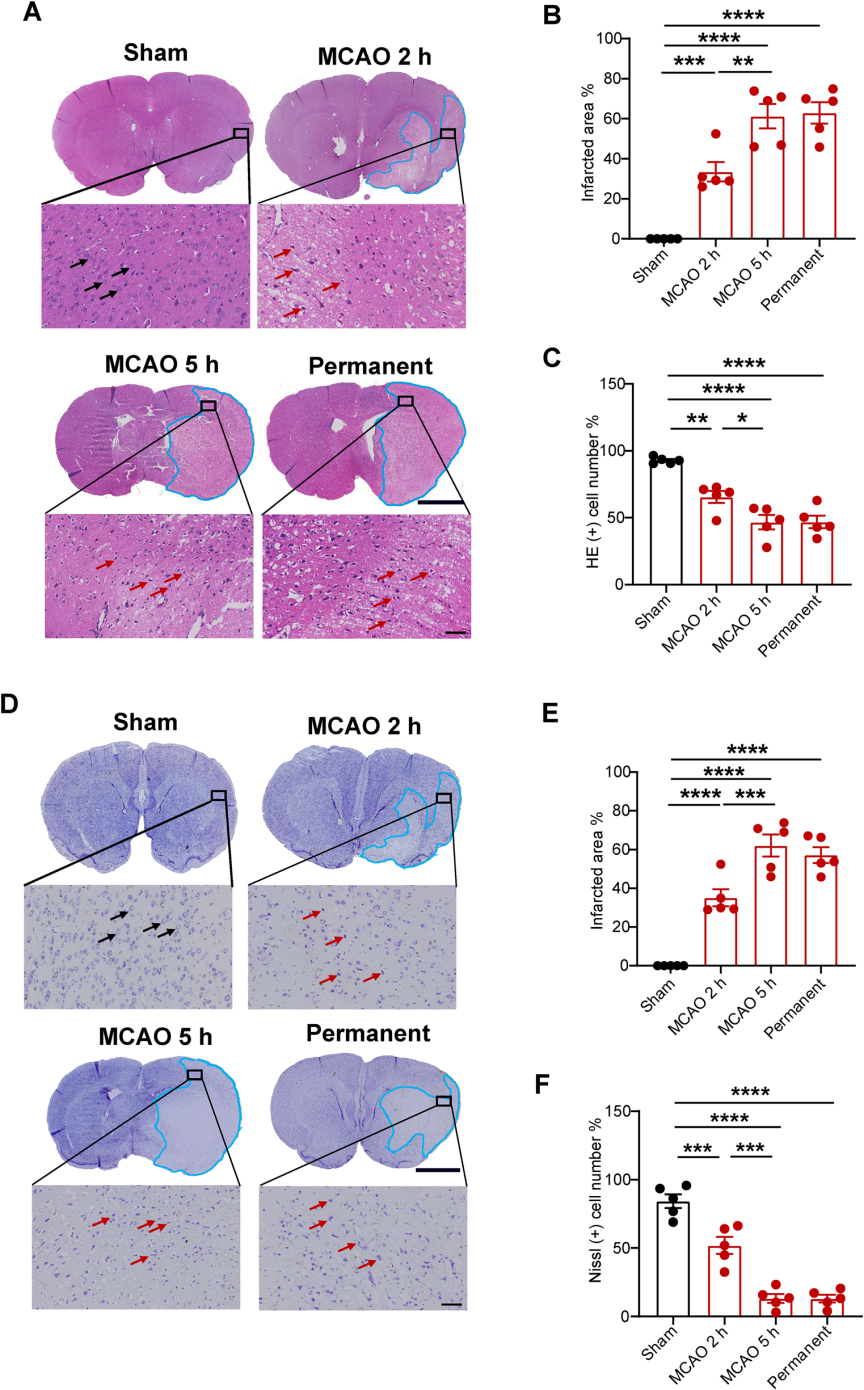
The neuronal injury in the ischemic penumbra was found to be exacerbated by thrombectomy beyond the time window. (A) The HE staining shows the area of HE (−) cells, represented by the blue curve. Scale bars = 2 mm. Black arrows represent the HE (+) cells, which had clear outlines and compact structures. Red arrows represent the HE (−) cells, which were arranged sparsely, had fuzzy outlines, and lacked structural organization. Scale bars = 50 µm. (B, E) Quantified infarct size by HE and Nissl staining, respectively. *n* = 5, data are presented as the mean ± S.E.M. **p < 0.01, ***p < 0.001, ****p < 0.0001, one‐way ANOVA followed by Tukey test. (C, F) The percentage of intact neuronal cells (HE+ and Nissl+) in the ischemic penumbra, respectively. *n* = 5, data are presented as the mean ± S.E.M.*p < 0.05, **p < 0.01, ***p < 0.001, ****p < 0.0001, one‐way ANOVA followed by Tukey test. (D) The Nissl staining shows the area of Nissl (+) cells, represented by the blue curve. Scale bars = 2 mm. Black arrows represent the intact neurons with full cell bodies. Red arrows represent the injured neurons with shrunken cell bodies accompanied by shrunken and pyknotic nuclei.

To investigate the cause of disparity in prognosis, the extent of neural injury of MCAO rats both within and beyond the time window for thrombectomy was examined using HE and Nissl staining. The injured cell showed a pale HE staining (HE−) that revealed poorly defined boundaries and cellular body atrophy, as well as nucleus atrophy and contraction, while the HE (+) exhibited clear outlines and compact structures and an intact nucleolus (Figure 4A). The infarcted area showed a substantial increase, with the most significant increase observed in the MCAO 5‐h group (Figure 4B,E). A significant reduction was observed in the percentage of HE (+) cells present in the ischemic penumbra, particularly evident in the MCAO 5‐h group (Figure 4C). As shown in Figure 4D, Nissl staining (Nissl+) revealed intact neurons characterized by plump cell bodies, whereas injured neurons exhibited shrunken cell bodies accompanied by condensed and pyknotic nuclei. A significant decrease was observed in the percentage of Nissl (+) cells present in the ischemic penumbra, particularly evident in the MCAO 5‐h group (Figure 4F). Reperfusion injury caused by thrombectomy beyond the therapeutic time window in rats may exacerbate neuronal cell injury in the ischemic penumbra, ultimately leading to more severe neurological impairment.

### 3.5 Reperfusion Injury From Thrombectomy Beyond the Time Window Could Worsen BBB Damage

**FIGURE 5:**
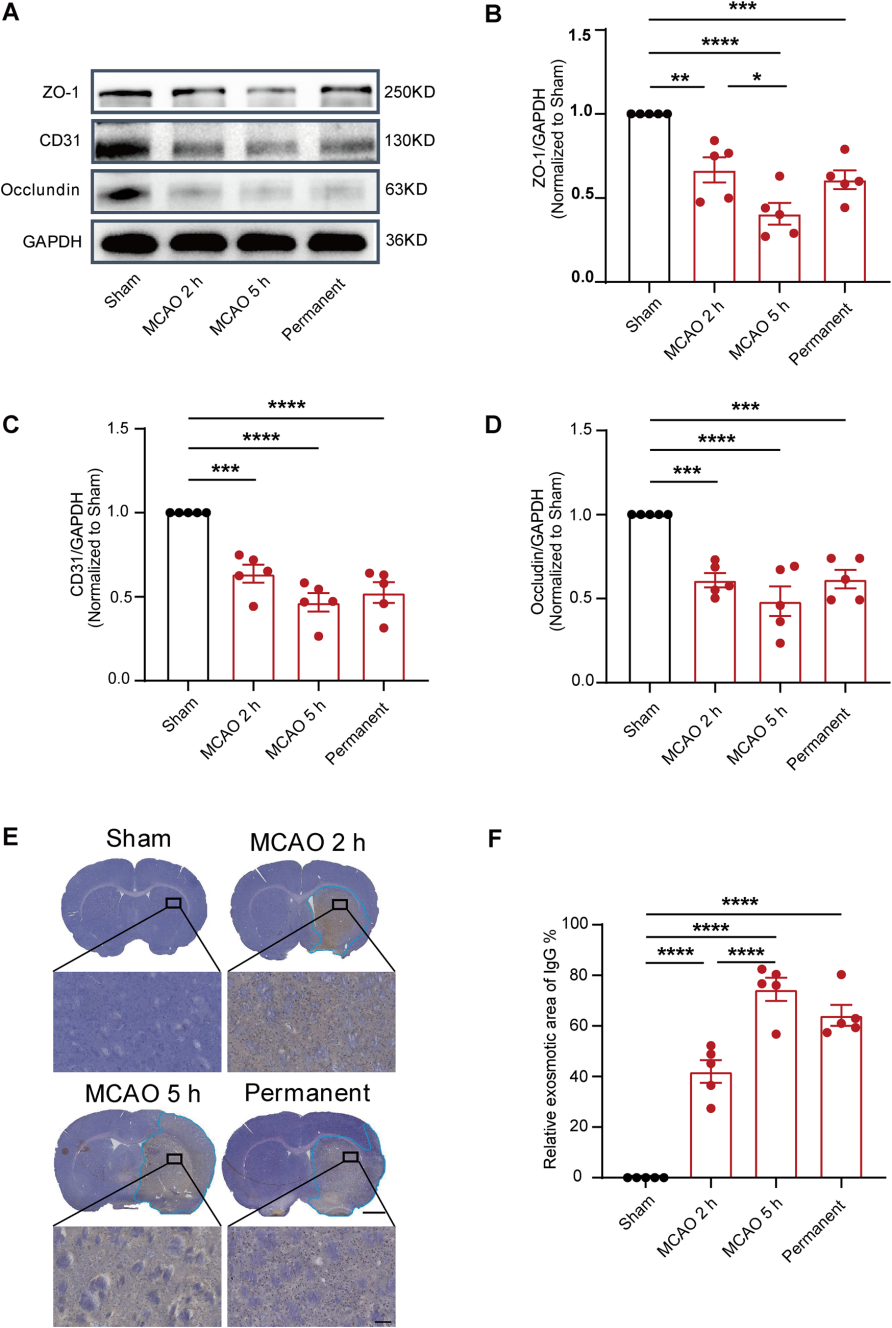
Reperfusion injury from thrombectomy beyond the time window could worsen both the structural and functional integrity of the BBB. (A) Immunoblotting showed the ZO‐1, CD31, and occludin proteins expression in the core infarction area 24 h after operation. (B–D) Quantitative analysis of the ZO‐1, CD31, and occludin protein levels relative to GAPDH (normalized to the sham group). *n* = 5, data are presented as the mean ± S.E.M. *p < 0.05, **p < 0.01, ***p < 0.001, ****p < 0.0001, one‐way ANOVA followed by Tukey test. (E) Immunohistochemical staining of IgG protein. The blue curve area represents the infiltration of IgG protein into brain tissue via blood vessels. (F) Quantify the area of IgG protein infiltrated by blood vessels into the brain tissue. *n* = 5, data are presented as the mean ± S.E.M. ****p < 0.0001, one‐way ANOVA followed by Tukey test.

To further investigate the pathological mechanisms of ischemia‐reperfusion injury, BBB function and inflammatory factors were evaluated in MCAO rats within and beyond the time window for thrombectomy. The molecular markers ZO‐1, CD31, and Occludin were utilized to define the structural components of the BBB (Jiao et al. 2011; Sugiyama et al. 2023; Y. Zhang et al. 2021). Western blot analysis revealed that the expression levels of the above three proteins significantly decreased in the MCAO 2‐h group, MCAO 5‐h group, and permanent group. Notably, the decrease of these molecules was more pronounced in the MCAO 5‐h group compared to the MCAO 2‐h group (Figure 5A–D). The plasma IgG protein was employed as a molecular marker for evaluating BBB permeability. Immunohistochemical staining revealed that substantial IgG protein crossed the BBB into brain tissue in the MCAO 2‐h group and MCAO 5‐h group, as well as the permanent group. In particular, the IgG protein levels in the MCAO 5‐h group were significantly elevated compared to those in the MCAO 2‐h group, indicating a more pronounced disruption in BBB permeability in rats undergoing thrombectomy beyond the therapeutic time window (Figure 5E,F). These findings indicate that reperfusion injury following thrombectomy beyond the time window may lead to an exacerbation of damage to the functional capacity of the BBB.

### 3.6 Reperfusion Injury From Thrombectomy Beyond the Time Window Increased Proinflammatory Factor Expression Levels

**FIGURE 6:**
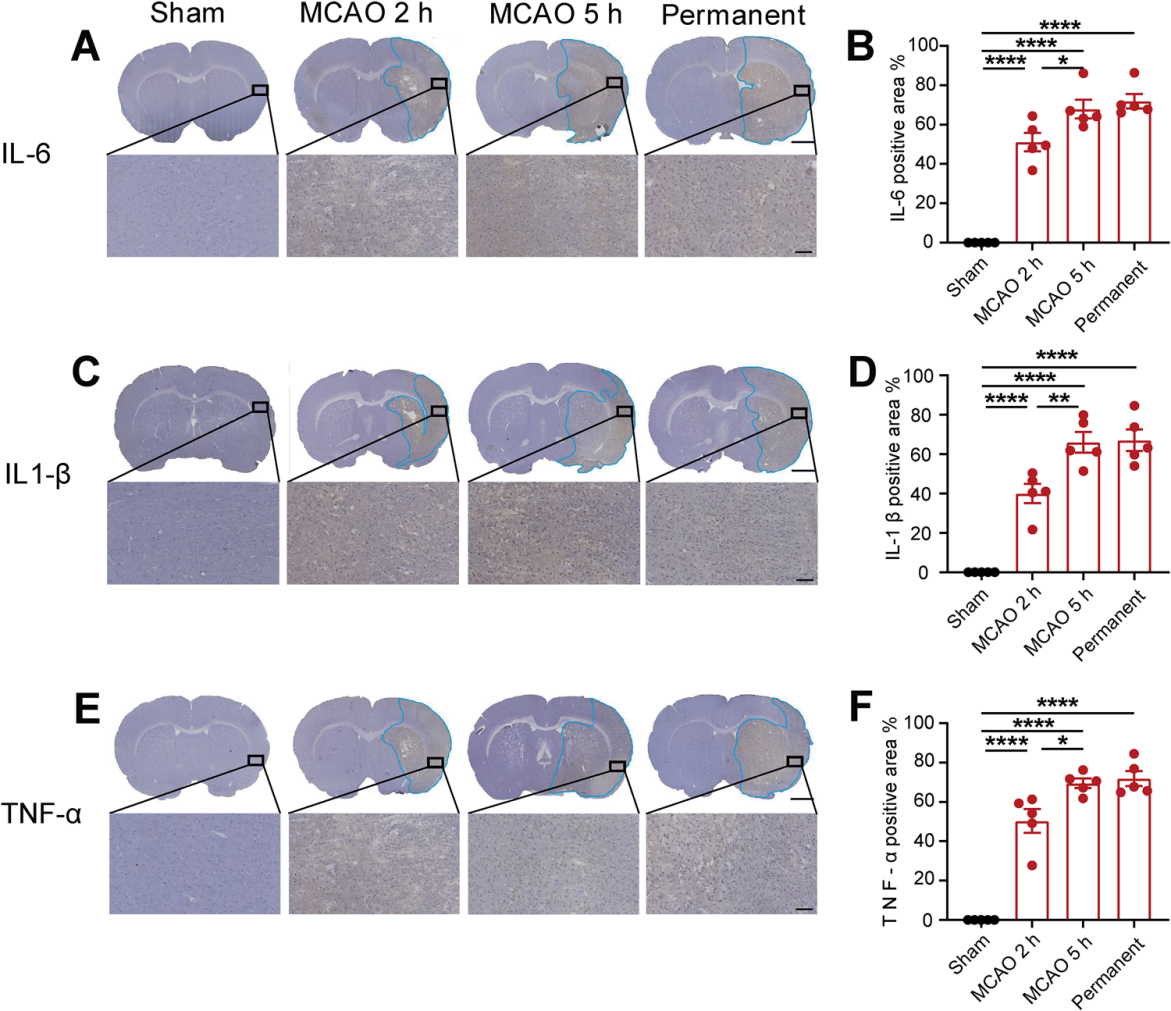
Reperfusion injury from thrombectomy beyond the time window may increase proinflammatory factor expression levels. (A, C, E) Immunohistochemical staining was performed to assess the protein levels of IL‐6, IL‐1β, and TNF‐α. The blue curve area indicated an upregulation in the expression levels of inflammatory factors, scale bars = 100 µm. (B, D, F) Quantify the area of increased expression levels of inflammatory factors. *n* = 5, data are presented as the mean ± S.E.M. *p < 0.05, **p < 0.01, ****p < 0.0001, one‐way ANOVA followed by Tukey test.

Malignant brain edema associated with ischemia‐reperfusion injury beyond the time window is often accompanied by an abnormal increase in inflammatory factors, which partially contributed to the disruption of BBB permeability (Candelario‐Jalil et al. 2022). In the ischemic area, the distribution and changes of inflammatory factors, including IL‐6, IL‐1β, and TNF‐α, were observed by immunohistochemical staining to assess the inflammatory response of rats subjected to varying durations of ischemia. Immunohistochemical staining revealed that the positive areas of three inflammatory factors increased significantly in the MCAO 2‐h group, MCAO 5‐h group, and permanent group. Notably, the areas of positive staining of these factors were larger in the MCAO 5‐h group compared to the MCAO 2‐h group (Figure 6). Therefore, the increased expression levels of inflammatory factors may contribute to the pathogenesis of brain edema after thrombectomy beyond the therapeutic time window.

## 4 Discussion

The data of the present study revealed that neurological impairment in rats is aggravated with the extension of ischemia time, while the therapeutic time window for thrombectomy in rats is likely to be within 4 h after MCAO. Compared with thrombectomy within the time window, the prognosis for MCAO rats undergoing thrombectomy beyond the time window was notably worse. Reperfusion injury caused by thrombectomy beyond the therapeutic time window in rats may exacerbate neuronal cell injury in the ischemic penumbra, further damage the functional capacity of the BBB, and increase expression levels of inflammatory factors, ultimately leading to more severe neurologic deficits.

Stroke is the second leading cause of disability and mortality globally, and AIS is the most prevalent type of stroke, accounting for approximately 60%–80% of all stroke cases (L. Liu, Chen, et al. 2020). In recent years, the therapeutic time window for mechanical thrombectomy in patients with AIS has been prolonged to 24 h, a substantial increase from the original guideline of 6 h (Campbell and Khatri 2020). Substantial clinical evidence has demonstrated that the outcomes of thrombectomy possessed a time‐dependent nature, wherein a reduction in favorable outcomes was closely correlated with the prolonged duration of reperfusion (Qureshi et al. 2014). In this study, although the neurological function score decreased to the lowest point at 4 h after ischemia‐reperfusion and remained stable, a significant increase in mortality was observed at 5 h after ischemia‐reperfusion, indicating that thrombectomy within 4 h following MCAO is the therapeutic time window in rats. Previous studies of MCAO in rats have primarily focused on ischemic reperfusion within the time window of 2 h (Fang et al. 2010; Khamchai et al. 2020; Pretzsch et al. 2022). In our study, we found that the damage and mortality rate were higher with reperfusion after a 5‐h ischemic beyond the time window, compared to a 2‐h ischemic within the time window. Additionally, the long‐term prognosis was also notably worse.

The application of endovascular thrombectomy therapy has significantly enhanced the rate of postoperative vascular recanalization for AIS caused by acute large vessel occlusion, reaching 70%–80% or even surpassing 90% (Heit et al. 2021). However, the rate of favorable clinical outcomes after thrombectomy was limited to 50% to 60%, which did not match the high rate of postoperative recanalization (Heit et al. 2021). In the study of MCAO ischemia‐reperfusion in rats, we also observed a similar phenomenon. LSCI imaging showed that CBF almost rose to the normal level after thrombectomy at various time points. However, the survival rate of rats with thrombectomy beyond the time window decreased to 62.5%, suggesting that mere physical vascular recanalization alone cannot improve the prognosis of stroke. But LSCI is limited to monitoring CBF on the surface of the cortex, possibly owing to technical limitations, and the results of magnetic resonance angiography or color Doppler flow imaging may provide more convincing.

It has been reported that the pathological mechanism might be attributed to exacerbated damage to neural cells and the functional capacity of the BBB caused by reperfusion injury during large vessel revascularization for thrombectomy in the acute stage (M. Liu, Xu, et al. 2020). In this study, HE and Nissl staining revealed a significant decrease in the percentage of structurally intact neuronal cells within the ischemic penumbra in the MCAO 5‐h group, as the “out‐of‐time‐window” group, and markedly lower than the MCAO 2‐h group (“within‐time‐window” group). It is suggested that the ischemia‐reperfusion injury beyond the time window of MCAO rats may be attributed to the aggravated injury of neuronal cells in the ischemic penumbra area, leading to impaired nerve function and poor prognosis.

The pathological process of CIRI involves the interplay of oxidative stress, calcium overload, inflammation, BBB injury, apoptosis, autophagy, microvascular dysfunction, and other biological mechanisms, collectively driving disease progression (Bai and Lyden 2015). The initiation of oxidative stress is initiated by a rapid surge in the supply of oxygen molecules during reperfusion, leading to an extensive generation of reactive oxygen species (ROS) (Jung et al. 2010). Thereinto, reperfusion injury‐induced cellular damage activated the immune response and initiated an inflammatory reaction (C. Yang et al. 2019). Inflammatory factors released by inflammatory cells, including macrophages and microglia, exacerbate brain tissue injury (C. Yang et al. 2019). Additionally, reperfusion‐induced production of ROS and inflammatory factors also inflicts damage on the BBB, leading to increased permeability. Impairment of the BBB integrity not only contributes to brain tissue edema but also exacerbates cellular injury (Cui et al. 2022). During this process, ischemia‐reperfusion also triggers intracellular apoptosis and autophagy pathways; while these mechanisms facilitate the clearance of damaged cells, their excessive activation can exacerbate cell death. (Q. Zhang et al. 2022). Finally, the increased microvascular permeability, thrombosis, and vascular inflammation that occur during reperfusion impede normal blood flow, leading to sustained ischemia in specific brain regions. (Guo et al. 2023). In the context of CIRI, these biological mechanisms collectively influence the interplay between neurons and glial cells, including astrocytes and microglial cells, thereby exacerbating neural damage and functional deterioration (Guo et al. 2023; Jurcau and Simion 2021; C. Yang et al. 2019). Therefore, employing a diverse range of molecular biological techniques, we observed a significant reduction in the protein levels of BBB‐associated molecular markers (ZO1, CD31, and Occludin) for MCAO rats that underwent thrombectomy beyond the time window (Jiao et al. 2011; Sugiyama et al. 2023; Y. Zhang et al. 2021). It suggests that impairment of the BBB may constitute one of the primary factors contributing to compromised neurological function. As a critical pathway for regulating substance transport in the central nervous system, impairment of the BBB could lead to an influx of peripheral immune cells into the central nervous system, triggering activation of inflammation‐related signaling pathways, secretion of various inflammatory factors, and ultimately neural cell damage (Jurcau and Simion 2021; C. Yang et al. 2019). We employed a range of neuroinflammatory markers (IL‐6, IL‐1β, TNF‐α) (Guo et al. 2023) and observed significant increases in the beyond time‐window ischemia‐reperfusion group, thereby corroborating this perspective.

Currently, the clinical benefits of thrombectomy beyond the time window remain controversial, potentially influenced by a multitude of factors. Notably, among patients with a favorable perfusion profile beyond 24 h, approximately 20% did not undergo thrombectomy within this timeframe, which may result in delayed infarct expansion and a worsened prognosis (Christensen et al. 2019). However, in patients with a mismatch between the severity of clinical defects and infarct volume, thrombectomy shows superior outcomes in terms of functional independence and disability compared to standard care alone (Nogueira et al. 2018). Therefore, timing of treatment, imaging assessment, and effective management of complications are crucial factors in achieving a favorable prognosis following thrombectomy beyond the time window. Additionally, the assessment of interventional therapy should also include the reperfusion injury that may occur following thrombectomy.

There are some limitations in our present study. Firstly, we employed LSCI to monitor CBF recovery on the infarct side; nevertheless, LSCI solely captures vessels on the cortical surface while disregarding subcortical vessels. Moreover, we only assessed changes in brain blood flow within 24 h post‐MCAO and did not conduct longer‐term follow‐up. In future investigations, alternative techniques such as Doppler ultrasound or MRA could replace LSCI and enable extended monitoring periods. Secondly, our exploration was limited to a finite time point and relied solely on a rat model for result verification, which may not fully represent the actual process of thrombolysis following human cerebral infarction. Further experiments should be conducted using other animal models. Lastly, although our explored mechanism is relatively simplistic, subsequent studies should undertake an in‐depth analysis of the ischemia‐reperfusion injury mechanism. Despite being preliminary and imperfect in nature, this study's design, based on this line of thinking, establishes a foundation for further exploration of ischemia‐reperfusion time windows in animal experiments and represents the first investigation into such a time window.

In conclusion, our study suggests that reperfusion injury during thrombectomy beyond the time window leads to BBB disruption, increased inflammatory products, and ultimately a marked decline in neural function scores. However, there is no significant correlation with infarct volume, indicating that this reperfusion injury may be a comprehensive pathological process involving the entire brain. Nevertheless, further experimental confirmation is still required.

## Author Contributions

**Yuting He**: data curation, visualization, writing – original draft, validation, supervision, investigation, formal analysis, methodology. **Pengcheng Huang**: supervision, project administration, methodology. **Shumeng Li**: methodology, investigation. **Jing Lin**: methodology, supervision. **Xiao Ren**: methodology, visualization. **Ying Xiong**: methodology, formal analysis. **Yuanjian Yang**: resources, software, project administration, supervision. **Daojun Hong**: writing – review and editing, conceptualization, resources, funding acquisition.

## Ethics Approval

The research was approved by the ethics committee of the First Affiliated Hospital of Nanchang University (Permission CDYFY‐IACUC‐202312QR004).

## Conflicts of Interest

The authors declare no conflicts of interest.

## Peer Review

The peer review history for this article is available at https://publons.com/publon/10.1002/brb3.70713

## Supporting information



Table S1. Garcia JH Neurological Scoring Criteria for Rats.
Fig S1. Garcia JH Neurological Scoring Criteria for Rats.

## Data Availability

Data will be made available on request.
