# Supplementary material for: Exploration of the Therapeutic Time Window for Thrombectomy in Rat Models of Middle Cerebral Artery Ischemia‐Reperfusion
Source: Brain Behav. 2025 Aug 4;15(8):e70713. doi: 10.1002/brb3.70713 (PMC12321963; doi:10.1002/brb3.70713)
Supplement: Supplementary file 1 — Table S1. Garcia JH Neurological Scoring Criteria for Rats. Fig S1. Garcia JH Neurological Scoring Criteria for Rats. [file BRB3-15-e70713-s001.docx]

| Text | 0 | 1 | 2 | 3 |
| --- | --- | --- | --- | --- |
| Spontaneous activity (Observe in the cage for 5 minutes). | No movement | Barely moves | Move and touching at least one side of cage | Moving and touching at least three side of cage |
| limb symmetry  (Observe four limbs after rats suspended) | Opposite limb: no movement | Opposite limb: slight movement | Opposite limb: slower movement than right side | Symmetrical on both sides. |
| Symmetry forelimbs  (Lift the tail to observe forelimb movement) | Opposite forelimb: no outreaching | Opposite forelimb: slight movement to outreach | Opposite forelimb: less movement to outreach compared to the same limb | Symmetry outreach |
| Cage climbing experiment | - | Falls to climb | Opposite forelimb is weak | Normal climbing |
| Body proprioception | - | Opposite side unresponsive | The opposite side stimulus response was slower than the same side | Both side reactions are the same |
| response to vibrissae touch | - | Opposite side unresponsive | The opposite side stimulus response was slower than the same side | Both side reactions are the same |

**Table S1.** [Garcia JH Neurological Scoring Criteria for Rats](https://www.researchgate.net/figure/Garcia-JH-Neurological-Scoring-Criteria-for-Rats_tbl1_363598258).


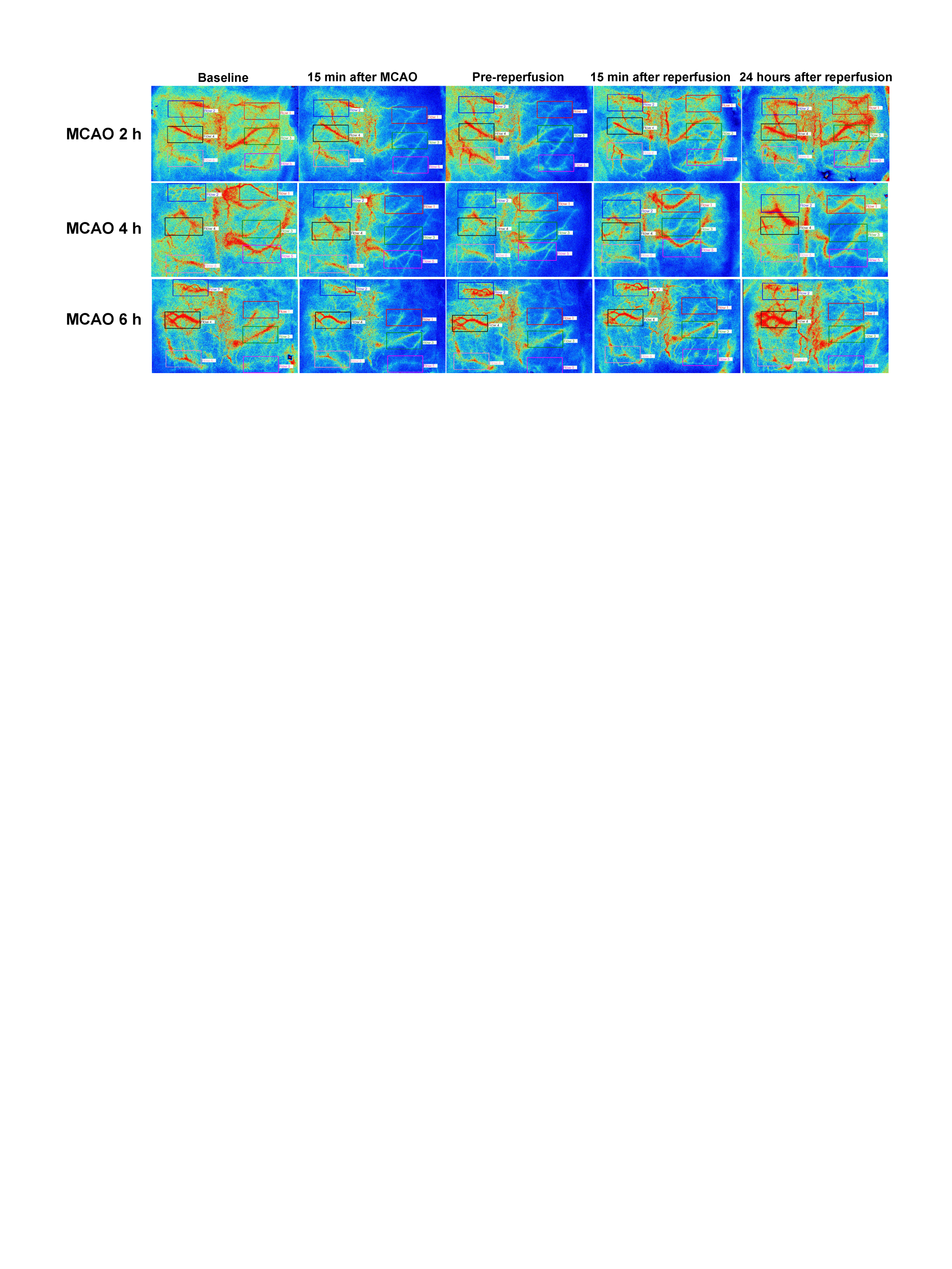


**Fig S1.** [Garcia JH Neurological Scoring Criteria for Rats](https://www.researchgate.net/figure/Garcia-JH-Neurological-Scoring-Criteria-for-Rats_tbl1_363598258).
